# The Use of Virtual and Augmented Reality in Anatomy Teaching

**DOI:** 10.15694/mep.2019.000077.1

**Published:** 2019-04-05

**Authors:** Amy Heather, Tudor Chinnah, Vikram Devaraj

**Affiliations:** 1 University of Exeter

**Keywords:** Medical education, Electronic resources, Virtual reality, Augmented reality, Anatomy, Technology Enhanced, Head-mounted display, Teaching and Learning, Innovative

## Abstract

This article was migrated. The article was marked as recommended.

**Background** - There is a demand for new and efficient tools to teach anatomical sciences. Rapid developments in virtual reality (VR) and augmented reality (AR) mean educational use of the technology is becoming increasingly viable. However, uptake of this technology in anatomy teaching is still limited. This brief review aims to examine the effectiveness of VR/AR in anatomy teaching and includes evaluation of: head mounted devices (HMDs), stereoscopic projectors and screens, AR Magic Mirrors and AR Magic Books.

**Methods** - PubMed, Scopus and Google Scholar were searched for relevant articles from 2013 to 29 ^th^ June 2018.

**Results** - Students’ academic performance was equal to or better than control methods for all four types of technology. All studies found high levels of student satisfaction for VR/AR teaching methods.

**Discussion** - Various confounding factors and the large heterogeneity between studies are likely to have a major impact on results. Further research into the depth and longevity of learning in the different teaching methods, as well as their cost-effectiveness, would be beneficial for prospective institutions.

## Introduction

In recent years, there has been growing interest in the use of virtual reality (VR) and augmented reality (AR) in anatomy education. This follows a decline in overall anatomy teaching time, as well as reductions in cadaver-based teaching and available laboratory hours ( Drake *et al.*, 2009). This decline in cadaver usage has created a demand for new and efficient tools to teach anatomical relationships.

Immersive and interactive 3D models can be displayed to students using VR/AR technologies. In VR, a user is fully immersed and feels present in a virtual environment. In AR, virtual objects (like anatomical models) are superimposed onto the user’s view of the real world. Models can be displayed on an individual basis through devices including mobiles, desktops and head-mounted devices (HMDs), and to wider audiences with stereoscopic projectors and screen-based AR systems.

Several institutions have trialled and used VR/AR technology as part of a multimodal, multimedia approach to anatomy teaching and learning. However, uptake of the technology is still limited. This can be due to concerns regarding the purchase cost and software development, as well as uncertainty over usability or efficacy of the technology. This brief review aims to examine the effectiveness of VR/AR in anatomy teaching, both in terms of academic results and student perceptions.

## Methods

A literature review was conducted using PubMed, Scopus and Google Scholar. Search terms included “virtual reality”, “augmented reality”, “mixed reality”, “head mounted device”, “anatomy” and “students”. To maintain temporal validity, papers prior to 2013 were excluded. The last search was conducted on 29 ^th^ June 2018.

## Results

The characteristics of the included studies (n=9) are summarised in Appendix 1.

### Head Mounted Devices

HMDs aim to create a sense of presence by realistically immersing the user in a shifting virtual environment displayed on head-mounted screens.

In this review, all three studies consulted found no significant differences in the post-test results of HMDs compared with the control alternatives: 2D images ( Ekstrand *et al.*, 2018), online textbooks ( Stepan *et al.*, 2017), AR tablets, and conventional tablets ( Moro *et al.*, 2017). Significant improvement between pre- and post-tests was observed in one study ( Ekstrand *et al.*, 2018) but not in another ( Stepan *et al.*, 2017). Adverse side effects were measured using a questionnaire in one study, and they found the VR group using HMDs experienced higher levels of side effects - including significantly more dizziness, blurred vision and general discomfort - than the AR tablet or tablet groups ( Moro *et al.*, 2017).

In student perception surveys, HMDs received significantly higher ratings than online textbooks for motivation, attention, confidence, satisfaction, interest and usefulness, but there was no significant difference for relevance or ease of use ( Stepan *et al.*, 2017). No significant differences were found between HMD and AR/tablet groups, though all the teaching methods had high ratings on how understandable or enjoyable the technology was and its usefulness as a supplementary teaching method ( Moro *et al.*, 2017). Compared to 2D images, HMDs received higher ratings for improved confidence and future use in teaching ( Ekstrand *et al.*, 2018).

### Stereoscopic Projectors and Screens

Stereoscopy is the display of different images to each eye, creating a sense of depth. This can enable more effective perception and understanding of spatial relationships in anatomy. It is utilised in presentations to large audiences through specialist projectors and filters, requiring the users to wear polarising glasses ( Kockro *et al.*, 2015; Cui *et al.*, 2017).

Students using a 3D stereoscopic model or 2D images both significantly improved in results between pre- and post-tests (p<0.001), with 3D stereoscopy achieving significantly higher post-test results (p=0.0033) ( Cui *et al.*, 2017). In the 3D stereoscopic group, students with low spatial ability performed as well as those with high (p=0.0899), but in the 2D group, they had significantly lower scores than those with high spatial ability (p=0.0259) ( Cui *et al.*, 2017). However, a study ( Kockro *et al.*, 2015) on students who received a 2D or stereoscopic 3D presentation on neuroanatomy reported no significant differences in post-test results ( Kockro *et al.*, 2015). Alternative research ( de Faria *et al.*, 2016) found that students instructed using 3D stereoscopic or 3D non-stereoscopic models performed significantly better in post-tests than those instructed using standard 2D images (p<0.05). However, there was no significant difference between stereoscopic and non-stereoscopic scores (p>0.05) ( de Faria *et al.*, 2016).

All three of the studies reviewed received positive feedback ( de Faria *et al.*, 2016; Cui *et al.*, 2017) and ratings ( Kockro *et al.*, 2015; Cui *et al.*, 2017) in student perception surveys. For one of the studies ( Kockro *et al.*, 2015), this is included significantly higher ratings from the 3D stereoscopic group for spatial understanding, future teaching, effectiveness and enjoyability than the 2D group (p<0.01) ( Kockro *et al.*, 2015). However, there were some reports of eyestrain and software limitations ( de Faria *et al.*, 2016).

### AR Magic Mirror

**Figure 1.**
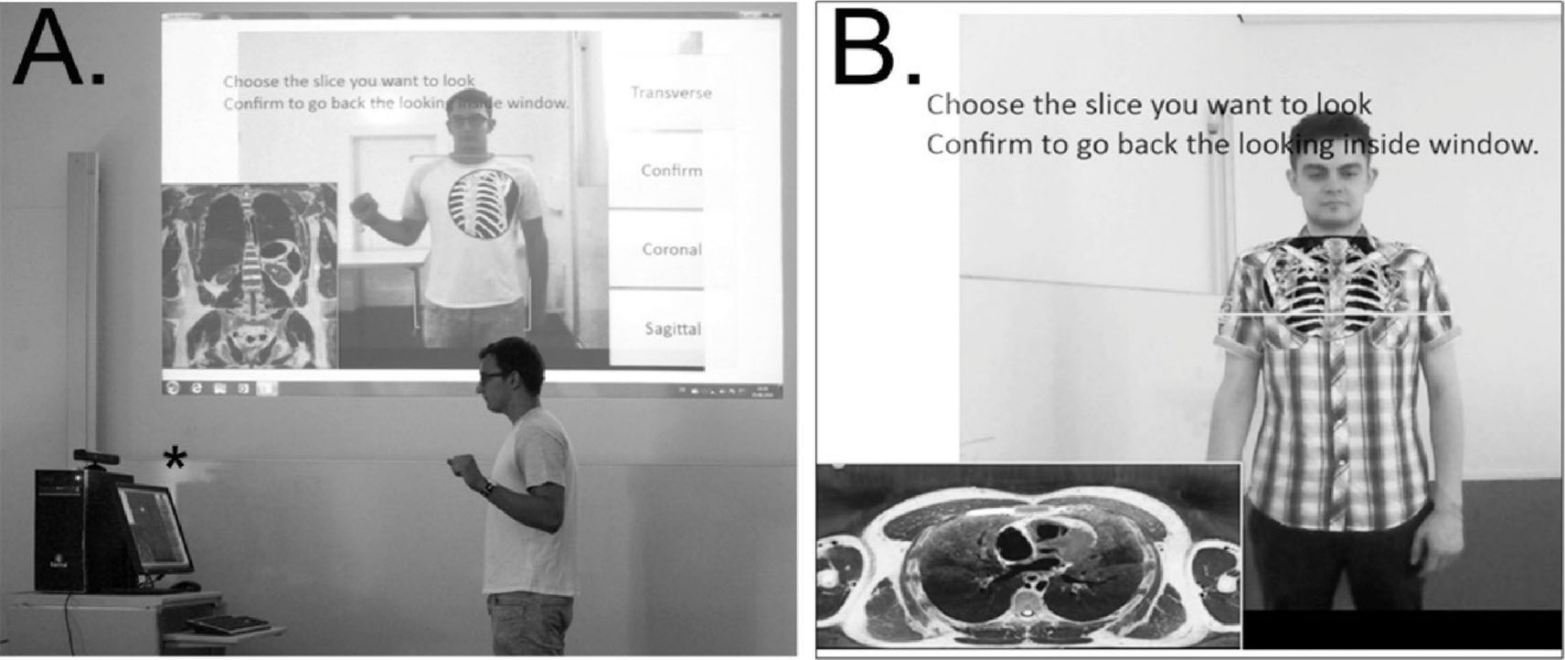
Students using AR Magic Mirror ( Kugelmann et al. 2018).

The AR Magic Mirror is a system where the user stands in front of a screen displaying their mirror image, whilst virtual images are superimposed onto them on the screen (see Figure 1). In anatomy teaching, these are often radiological or anatomical sections. Through real-time tracking, the images align with the user to represent the precise location of structures within the user’s body ( Kugelmann *et al.*, 2018).

In a study ( Kugelmann *et al.*, 2018) using the AR Magic Mirror to teach anatomy, a survey was administered to investigate student perceptions of teaching anatomy with the AR Magic Mirror. They received strong positive responses, notably for its usefulness in active learning and 3D understanding. Criticisms of the software revolved around technical problems and suggestions of more content ( Kugelmann *et al.*, 2018).

### AR Magic Book

Virtual objects can be superimposed onto the pages of an AR Magic Book when it is viewed through the screen of a suitable device, like a tablet or mobile. The physical book contains markings that devices can identify, track and augment content to. This content includes 3D models, animations and videos ( Küçük, Kapakin and Göktaş, 2016).

Students studying anatomy with an AR Magic Book performed significantly higher than those using a dissection video in a post-test (p<0.000) and had a significantly lower score distribution ( Ferrer-Torregrosa *et al.*, 2016). This is consistent with a study into a mobile AR (mAR) Magic Book, where the post-test performance of students using an mAR Magic Book was significantly higher than those using 2D images (p<0.05) and was associated with significantly lower cognitive load (p<0.05) ( Küçük, Kapakin and Göktaş, 2016).

Positive student perceptions of the AR Magic Book were found in both studies ( Ferrer-Torregrosa *et al.*, 2016; Küçük, Kapakin and Göktaş, 2016). This included a survey on motivation, autonomous learning and 3D understanding ( Ferrer-Torregrosa *et al.*, 2016), and interview responses regarding effectiveness, motivation and interest ( Küçük, Kapakin and Göktaş, 2016).

## Discussion

Reported research studies suggest that VR and AR are at least as effective as traditional anatomy teaching methods. However, this generalisation may be unreliable due to the large heterogeneity between studies. As there is a limited number of studies available, this review included various teaching methods that may not be directly comparable. Another confounding factor is the control method used. Whilst there is value in comparing VR/AR to one simple teaching method like 2D diagrams or textbooks, it can be more insightful to also compare it with multiple methods, like standard 3D physical and virtual models. This is demonstrated by an included study ( de Faria *et al.*, 2016) in their conclusion that results varied between 2D and 3D models, but not necessarily between stereoscopic and non-stereoscopic ( de Faria *et al.*, 2016). Considering the often-high costs of VR/AR hardware acquisition and software development compared with standard online 3D models, this would be valuable information for prospective institutions.

Results may have also been influenced by large variation in study topic, participant degree courses, prior knowledge of students, timing of the study (whether used to introduce or reinforce a topic), form of study (individual or group) and study time. Differences in model quality and features (such as the level of interactivity) may have also impacted results. Furthermore, for many studies, results may be unrepresentative due to small sample sizes.

The number of questions, as well as their difficulty and the presence of any repeats, could have also impacted results. Results offered restricted insight into learning as studies largely used short MCQ tests that reflect factual recall over other forms of understanding, such as that of 3D relationships. For more translatable and insightful findings, all studies would need pre-, post- and long-term recall tests.

Despite the wide heterogeneity, there is a consensus between studies regarding positive student perceptions of VR/AR. In all studies, surveys revealed high levels of student satisfaction - generally higher than control alternatives - demonstrating the benefits of VR/AR for student enjoyment and engagement. It can be observed that VR/AR had a notable impact on spatial understanding, motivation and interest. Other reported areas of benefit included autonomous learning, confidence, cognitive load and overall student perceptions.

These survey results may be subject to sampling bias as participants were often volunteers and so more likely to be motivated and engaged. There may also be author bias, as evaluated systems were often developed by or for the paper authors. The novelty of this new technology may also affect positive perceptions. Moreover, the five-point Likert scales generally used could produce response bias, whilst yes/no responses ( Küçük, Kapakin and Göktaş, 2016) are rather restrictive. Study responses are also limited when there are no reported control responses for comparison.

## Conclusion

Studies indicate that VR and AR are effective and capable resources for anatomy teaching in terms of both academic achievement and student satisfaction, supporting the use of VR and AR as supplements to current teaching methods. However, further research into the depth and longevity of learning would aid understanding of the extent of their long-term impact on academic achievement. Future investigations into cost-effectiveness would also be helpful.

## Take Home Messages

• There is a potential for use of VR/AR in anatomy teaching, although uptake has been limited.
• Studies found students’ academic performance was equal to or better than control methods for all four types of VR/AR technology.
• All studies found high levels of student satisfaction for VR/AR teaching methods.
• Minimal conclusions can be drawn on the true effectiveness of VR/AR due to limitations of studies. These include poor assessment of learning and failure to compare VR/AR against a range of other teaching resources.
• Further research is required into the depth and longevity of learning and the cost-effectiveness of VR/AR.

## Notes On Contributors

**Amy Heather**, 2nd Year BSc Medical Sciences Student, University of Exeter.

**Tudor Chinnah**, PhD, MScClinEd, MSc, BMedSc, SFHEA, Senior Lecturer, Human Anatomy & Clinical Education, Academic Lead, Human Anatomy, University of Exeter Medical School.

**Vikram Devaraj**, PGCE Clin Ed MBChB FRCS(Eng) FRCS(Plast), Senior Lecturer & Clinical Lead Anatomy, Consultant Surgeon.

## Appendices

**Appendix 1.** Characteristics of Included Studies

Study	Sample & Study Topic	Teaching Device & Participants Numbers	Study Time	Measurements
(Ekstrand et al. 2018)	64 medical students Neuroanatomy	**HMD** *3D model for HMD created by paper authors from MRI scans using commercial software. 2D images adapted from neuroanatomy textbook* 31 - HTC Vive 33 - 2D images on paper	12 minutes (plus 5-minute HTC Vive tutorial)	• Pre-test (22 questions) • Post-test (22 questions) • Retention test (44 questions, 5-9 days later) • Student perceptions survey
(Stepan et al. 2017)	66 medical students Neuroanatomy	**HMD** *3D model for HMD created from CT & MRI scans using commercial software* 33 - Oculus Rift 33 - Online textbook	20 minutes textbook, or 10 minutes VR and 10 minutes textbook	• Pre-test (10 questions) • Post-test (30 questions) • Retention test (15 questions, 8 weeks later) • Student perceptions survey including IMMS survey
(Moro et al. 2017)	59 students - biomedical, health science, medicine or other Skull anatomy	**HMD** *3D model for HMD and tablets created by paper authors using commercial software* 20 - Oculus Rift 17 - AR on tablet 22 - Tablet	10 minutes	• Post-test (20 questions) • Student perceptions survey • Adverse side effects survey
(Cui et al. 2017)	39 medical students Head and neck vasculature	**Stereoscopy** *3D model created using CTA data and commercial software* 18 - 2D snapshots of 3D model & radiology images 21 - 3D model on stereoscopic screen	20 minutes (plus 5 minute introductory lecture)	• Pre-test (15 questions) • Post-test (15 questions) • Mental rotation test (MRT) • Student perceptions survey
(Kockro et al. 2015)	169 medical students Neuroanatomy - ventricular system	**Stereoscopy** *3D model created from cranial MRI scans using commercial software* *2D images sourced from anatomy textbooks* 89 - 3D Dextrobeam stereoscopic projector 80 - 2D powerpoint	20 minutes	• Post-test (10 questions) • Student perceptions survey
(de Faria et al. 2016)	84 medical students Neuroanatomy - limbic system	**Stereoscopy** *3D model created from brain section photographs and developed using commercial software* 28 - 2D lecture 28 - Non-stereoscopic interactive 3D lecture 28 - Stereoscopic interactive 3D lecture	50-60 minutes	• Pre-test (10 marks) • Post-test (20 marks) • (3D only) Asked to describe advantages and disadvantages
(Küçük et al. 2016)	70 medical students Neuroanatomy - Medulla Spinalis	**mAR Magic Book** *mAR Magic Book created using commercial software* 36 - Conventional lecture with 2D images 34 - Conventional lecture with addition of MagicBook	5 course hours plus independent study	• Post-test (30 questions) • Cognitive Load scale • Student perceptions interview
(Kugelmann et al. 2018)	880 medical students Anatomy from radiology images	**AR Magic Mirror** *Created using radiological images and commercial software* 880 - AR Magic Mirror (though only received responses from 748)	Not stated	• Student perceptions survey
(Ferrer-Torregrosa et al. 2016)	177 students - medicine, physiotherapy & podiatry Extrinsic muscles of foot	**AR Magic Book** *AR Magic Book resource requested from LabHuman company by authors of paper* Notes with...60 - 2D images 52 - Dissection video 60 - AR Magic Book	Independent study in 2 week period	• Questionnaire on time spent studying • Post-test (10 marks) Student perceptions survey
