# Supplementary material for: The Use of Virtual and Augmented Reality in Anatomy Teaching
Source: MedEdPublish (2016). 2019 Apr 5;8:77. [Version 1] doi: 10.15694/mep.2019.000077.1 (PMC10712594; doi:10.15694/mep.2019.000077.1)
Supplement: Peer review history

# Reviewer report: Reviewer response for version 1

**DOI:** 10.21956/mep.19809.r29819

**Published:** 2019-04-15

**Authors:** Simonetta Ausoni

**Affiliations:** 1 Department of Biomedical Sciences- University of Padova- School of Medicine

This review has been migrated. The reviewer awarded 3 stars out of 5 I read the manuscript with great interest and I think it is important to host discussion on a issue that is involving many academic schools, medical and not, for the preparation of innovative technological tools for the study of anatomy. Having adequate feedback on the advantages and disadvantages of this approach and, above all, on the effects on learning anatomy is necessary. As other reviewers suggested, I also encourage a revision of the manuscript that should cover aspects that have not been highlighted or deeply discussed in this version.Like other reviewers have underlined, major points are:1. Please detail the methodology used in the review to select or exclude the papers;2. Detail, whenever possible, the characteristics of study groups mentioned in the original papers;3. Describe major results achieved by the use of innovative technologies. How were advantages or disadvantages quantified in the original papers?4. I also agree that the Table should be included in the text and that more images and schemes describing better each approach should be included5. A conclusion and reflection on how further research should be planned to provide adequate insights on the benefits of advanced technologies in learning anatomy should be included.

Reviewer Expertise:

NA

No decision status is available

# Reviewer report: Reviewer response for version 1

**DOI:** 10.21956/mep.19809.r29822

**Published:** 2019-04-12

**Authors:** Trevor Gibbs

**Affiliations:** 1 AMEE

This review has been migrated. The reviewer awarded 3 stars out of 5 Thank you for asking me to review this paperIt seems that there is a constant flow of new and innovative technologies to enhance student learning- the teaching and learning of anatomy is frequently at the front of this rise in interest so a paper that reviews the literature surrounding the subject seems a very good paper to be included in our toolbox of relevant literature.I was rather disappointed that the authors failed to explore in greater detail their approach to their search methodology, so that we could be assured that this was really an effective review.As the authors point out, and it maybe a reflection of the papers so far, that many of the evaluations of the various technologies are based upon student satisfaction, rather than usefulness in the process of learning and long term effects. I do think that this paper would be of limited value for our anatomy teachers.

Reviewer Expertise:

NA

No decision status is available

# Reviewer report: Reviewer response for version 1

**DOI:** 10.21956/mep.19809.r29817

**Published:** 2019-04-11

**Authors:** Prerna Agarwal

**Affiliations:** 1 Datta Meghe Institute of Medical Sciences

This review has been migrated. The reviewer awarded 3 stars out of 5 The paper is indeed in sync with the need of using technology for advancing education in the field of anatomy in view of the limited availability of cadavers for teaching it. The authors have highlighted the available resources for the same and discussed how previous studies weighed the use of VR/AR. But I have a few reservations, addressing which I think will significantly improve the scope of this review of the literature:1. What age/ course students were included in the studies reviewed here? The perspective and need of a senior student, may be one pursuing a post-graduation in a surgical branch having the to revisit anatomy, will be different from a first year student, who has just entered the field. 2. Like other reviewers have mentioned, including the table in the main body of the article will be more useful.

Reviewer Expertise:

NA

No decision status is available

# Reviewer report: Reviewer response for version 1

**DOI:** 10.21956/mep.19809.r29818

**Published:** 2019-04-10

**Authors:** Iain Keenan

**Affiliations:** 1 Newcastle University

This review has been migrated. The reviewer awarded 3 stars out of 5 Many thanks to the authors for this brief but welcome and interesting review of VR/AR in anatomy education. I would like to have seen a more detailed introduction to the paper, perhaps describing the scholarly basis and theoretical underpinnings of visual technology-enhanced learning approaches in anatomy.Like the other reviewers, I would like to have seen a more detailed description of the methodology and perhaps a systematic review approach utilised in future, which I would read with great interest. Due to the visual nature of the subject matter I would also like to have seen further images, and in colour. I also agree that the table would be more effectively presented within the main body of the article.

Reviewer Expertise:

NA

No decision status is available

# Reviewer report: Reviewer response for version 1

**DOI:** 10.21956/mep.19809.r29820

**Published:** 2019-04-05

**Authors:** Lee HangFu

**Affiliations:** 1 Windsor University School of Medicine

This review has been migrated. The reviewer awarded 3 stars out of 5 Thank you for the opportunity to read this manuscript titled "The Use of Virtual and Augmented Reality in Anatomy Teaching". The brief explanation of the VR/AR by the author is useful but superficial. Readers may benefit from the explanation on the Learning Objective content delivery methodology. It is an interesting review of the VR/AR teaching tool.

Reviewer Expertise:

NA

No decision status is available

# Reviewer report: Reviewer response for version 1

**DOI:** 10.21956/mep.19809.r29816

**Published:** 2019-04-05

**Authors:** P Ravi Shankar

**Affiliations:** 1 American International Medical University

This review has been migrated. The reviewer awarded 4 stars out of 5 Thank you for the invitation to review this interesting manuscript. The authors address an area of increasing importance in medical education. AR and VR technologies are widely used in Anatomy teaching-learning and have many advantages over traditional teaching-learning methods. The table provides a useful summary of the different studies included in the review. AR magic mirror and AR magic book were new concepts to me and the authors could describe these in greater detail. The authors could provide a more detailed description of the inclusion and exclusion criteria followed for the various studies included in the paper. The authors have described the limitations of their study. The paper will be of special interest to medical educators especially those teaching Anatomy.

Reviewer Expertise:

NA

No decision status is available

# Reviewer report: Reviewer response for version 1

**DOI:** 10.21956/mep.19809.r29821

**Published:** 2019-04-05

**Authors:** Ken Masters

**Affiliations:** 1 Sultan Qaboos University

This review has been migrated. The reviewer awarded 3 stars out of 5 This is an interesting review dealing with a rapidly emerging topic. The small number of included studies is indicative of the novelty of the teaching methods. While later reviews are sure to introduce issues and results not foreseen in this review, this is a useful starter to the field. And, as with all reviews, the authors are constrained by the quality of the original studies, and the data they present.I would like to make two recommendations for Version 2 of this paper:• Although this paper is not billed as a systematic literature review (SLR), it does appear to have elements of it. I would strongly recommend that the authors flesh out the description of their Methods, and give the types of details that one would find in an SLR, typically a diagram showing numbers on initial search, exclusions, inclusions, etc. This would dramatically improve the quality of the paper.• Appendix 1 contains a table that is an essential part of the paper. This table should not be in the appendix, but should be placed into the body of the paper. An appendix should really only be used for material that is nice to have, in case anybody is interested, or to not interrupt the flow of the paper. In this case, however, the table in Appendix 1 is a significantly important portion of the Results, and should be inserted there.

Reviewer Expertise:

NA

No decision status is available
